# Durability and Mechanical Performance of PMMA/Stone Sludge Nanocomposites for Acrylic Solid Surface Applications

**DOI:** 10.3390/polym9110604

**Published:** 2017-11-12

**Authors:** Samah EL-Bashir, Nouf Althumairi, Naser Alzayed

**Affiliations:** 1 Department of Physics & Astronomy, Science College, King Saud University, Riyadh 11461, Saudi Arabia; alwafi399@hotmail.com (N.A.A.); nalzayed@ksu.edu.sa (N.A.); 2 Department of Physics, Faculty of Science, Benha University, Benha 13513, Egypt

**Keywords:** PMMA/clay nanocomposites, mechanical properties, acrylic solid surfaces

## Abstract

Acrylic solid surface sheets were prepared by mixing different kinds of stone sludge fillers (SSF) in Poly (methyl methacrylate) (PMMA) nanocomposites. PMMA nanocomposite syrups were made using free radical polymerization of methylmethacrylate (MMA), then two kinds of nanofillers were added, namely, hydrophilic nanosilica and clay Halloysite nanotubules (HNTs). Acrylic solid surface sheets were manufactured by mixing the syrups with SSFs. The morphology of the produced sheets was studied using optical, and Scanning Electron Microscopy (SEM) that revealed the uniform distribution of stone sludge in the polymeric matrix. The study of the physical properties showed promising mechanical performance and durability of PMMA/SSF nanocomposites for acrylic solid surface applications.

## 1. Introduction

Polymer nanocomposites are hybrid materials based on polymeric matrices filled with nanoscaled inorganic molecules. These materials can be tailored to combine the advantages of both inorganic materials (e.g., rigidity, stability) and organic polymers (e.g., flexibility, ductility, processability, low cost) [1,2,3]. Thus, the new composites usually contain special features of nanofillers leading to new materials with improved properties [4]. In contrast to traditional fillers, the little size of the nanofillers leads to a dramatic decrease of the polymer free volume and subsequently improves the mechanical properties [5,6,7]. This property may be due to the significant effect of the nanocomposite’s phase morphology which depends on the interphase interaction [8]. The present study presented a new type of acrylic solid surface (artificial marble) that is based on PMMA nanocomposite matrices filled with stone sludge fillers (SSF) obtained from the remains of natural stones such as marble, granite, and basalt. Our group offered a green solution to the environment by making use of stone sludge which has no economic value and causes the pollution of the environment [5,6,7]. The innovated acrylic solid surfaces have overcome the disadvantages of natural stones such as color staining, water absorption, heavyweight, expensive cost, hard processing and poor resistance to weak acids [5,7,9]. These products have additional advantages over natural stones such as thermal and electrical insulation, non-porosity which prevents the growth of bacteria and mottling [6,10]. In addition, the prepared acrylic solid surface samples represent a cheap and durable replacement to the natural stones with promising thermal stability for outdoor decorative applications in hot countries like Saudi Arabia.

## 2. Experimental Techniques

### 2.1. Materials

**Figure 1:**
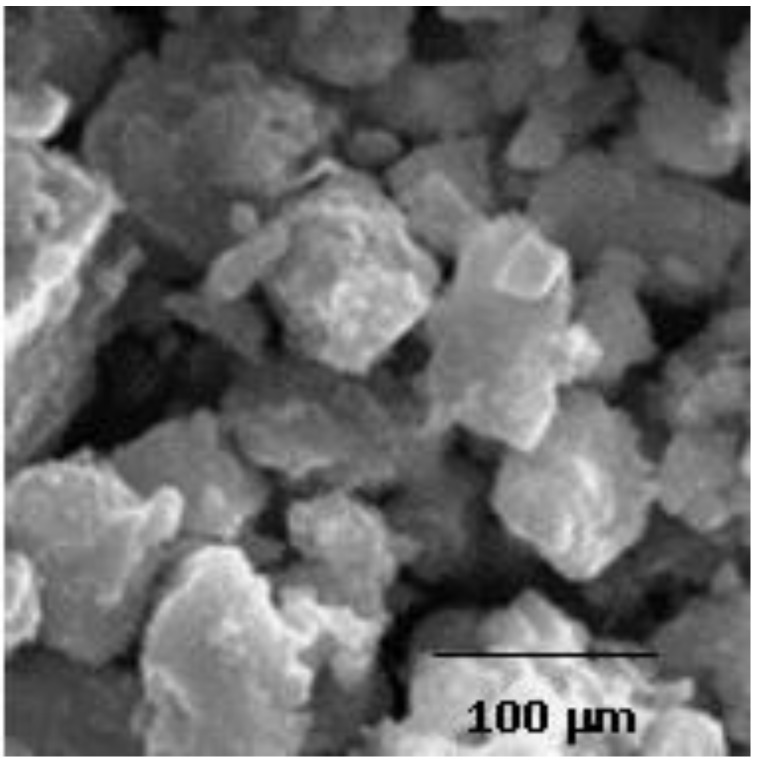
SEM of marble sludge, (typical image of all the investigated kinds of stone sludge fillers (SSF)).

Methyl methacrylate (MMA) monomer was obtained from (Merck, Darmstadt, Germany). Hydrophilic nanosilica of 7 nm diameter (Aldrich, St. Louis, MO, USA) was used as a thixotropic agent and anticorrosive material [11,12]. Nanoclay Halloysite nanotubules (HNTs) were purchased from (Aldrich), with an average tube diameter of 50 nm and inner lumen diameter of 15 nm [13]. Stone sludge fillers (SSF) were produced by successive sieving of the sludge of natural stones such as marble, basalt, and granite. The average particle diameter of SSF was about 50 μm as determined by SEM micrograph presented in Figure 1.

### 2.2. PMMA Nanocomposite Preparation 

The polymer syrup (PMMA-MMA) was prepared by adding 0.1 wt % of azobisisobutyronitrile (AIBN) initiator (DuPont, Wilmington, DE, USA) MMA. The monomer solution was refluxed at 100 °C under reduced pressure (10^−3^ bar) for one hour to increase the syrup viscosity and decrease the polymerization time [5,6,7]. The measured viscosity of the resulting syrup was 200 cP using a rotational viscometer (BROOK FIELD DV-II, LabX, Midland, ON, Canada). Then, 20 wt % of Alumina Trihydrate (ATH) (Albemarle Corp., Baton Rouge, LA, USA) was mixed with the syrup as anti-flammable and anti-corrosive material [5,6,7]. Next, Ethylene glycol dimethacrylate was added as a cross-linking agent at a concentration 0.5 wt %. Afterward, Hydrophilic nanosilica and nanoclay halloysite nanotubules (HNTs) were added as received at concentrations 2 wt % and 3 wt %. These concentrations were chosen in our previous work as the optimum for the enhancement of thermal stability and mechanical properties for PMMA nanocomposites [5]. The viscosity of PMMA-MMA syrup was equal to 800 cP and 1050 cP after adding nanosilica and nanoclay, respectively. This concentration was recorded to give the maximum improvement of the thermal stability and mechanical properties for preparing PMMA/clay HNTs nanocomposites [14]. The nanocomposite syrups of PMMA/SiO_2_ and PMMA/clay HNTs were mixed with different concentrations of SSF in the range (10–80 wt %). The mixtures were cast into rectangular tempered glass cells having dimensions 50 × 50 × 2 cm^3^ as described in detail in ref. [5].

### 2.3. Autoclave Polymerization Technique

The casting cells were placed in an autoclave in which the pressure and temperature can be adjusted to obtain the desired thickness of the prepared PMMA/stone nanocomposite. Firstly, the autoclave was evacuated from the air and then pressurized at 2 bars with nitrogen at 60 °C. Both temperature and pressure were maintained constant until the polymerization process is completed then the casting cells were cooled slowly to room temperature without changing the pressure. After that the pressure was reduced gradually to the normal atmospheric value, then the casting cells were put in an electric oven at 100 °C for 2 h for the final curing to complete the polymerization process of acrylic solid surface sheets.

### 2.4. Testing Methods

The mechanical properties were tested using the tensile machine (UNIVERSAL Tester AT 2.5KN 84-02, Messmer Instruments Ltd.) at a rate of 1 mm/min. Three-point bending test was carried out for the investigated PMMA/SSF nanocomposite slabs; the test was performed in accordance with ASTM standard (D7264) [15]. Impact strength measurements have been made according to (ASTM D 6110-2) [16], using Charpy impact tester (43-02 monitor/impact, Messmer Instruments Ltd.). The Rockwell hardness was evaluated to provide useful information about acrylic solid surface nanocomposites as engineering materials. This measurement is important to correlate with tensile strength, abrasion resistance, ductility, and other physical characteristics of PMMA/stone nanocomposites. It is also will be useful in the quality control by providing the selection of optimum composition for the investigated acrylic solid surfaces. The hardness of the samples was measured using Rockwell hardness testing machine (Wilson 2000T, Buehler, Lake Bluff, IL, USA) according to ASTM (E18-11) [17]. The abrasion resistance was tested according to ASTM (D1044-08e1) [18,19], using Taber abrasion tester (Messmer 5130, Messmer Instruments Ltd., Kent, UK). Water swelling test of PMMA/SSF nanocomposites was examined according to ASTM (D570-98 e1) [5,19]. The thermal conductivity of the samples was measured using thermal conductivity apparatus (PASCO TD-8561, PASCO, Roseville, CA, USA). The electrical conductivity σ_dc_ has been measured for all the nanocomposite samples using a conductivity meter (Model 1116 SLD, IJ Cambria Scientific Ltd., Llanelli, UK). Thermal stability of the prepared nanocomposite samples was measured using thermogravimetric analyzer apparatus (TGA-50H, Shimadzu, Kyoto, Japan). The samples were heated from room temperature to 1400 °C at a heating rate of 10 °C/min in a nitrogen atmosphere.

## 3. Results and Discussion

**Figure 2:**
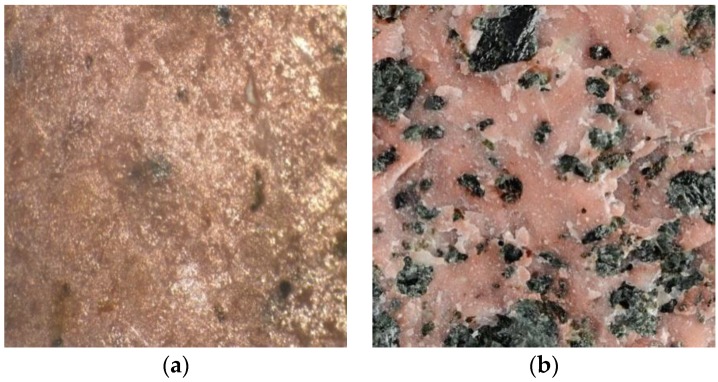
Surface Morphology (M × 35) of (**a**) Artificial Granite (60 wt % SSF/Poly (methyl methacrylate) (PMMA)/clay Halloysite nanotubules (HNTs)) compared to (**b**) natural Granite.

Figure 2 shows the cross-sectional view of granite based PMMA/SSF/SiO_2_ nanocomposite; the photograph illustrates that the granite sludge is well dispersed inside PMMA nanocomposite matrix causing the observed red color of PMMA/SSF/SiO_2_ nanocomposite. It is also noted that granite particles are completely coated and homogeneously distributed with an excellent physical adhesion between SSF and the polymer nanocomposite; similar images were obtained for marble and basalt fillers. This result is important for acrylic solid surface applications since the treatment with PMMA nanocomposites can efficiently overcome the porosity and surface roughness of granite sludge.

**Figure 3:**
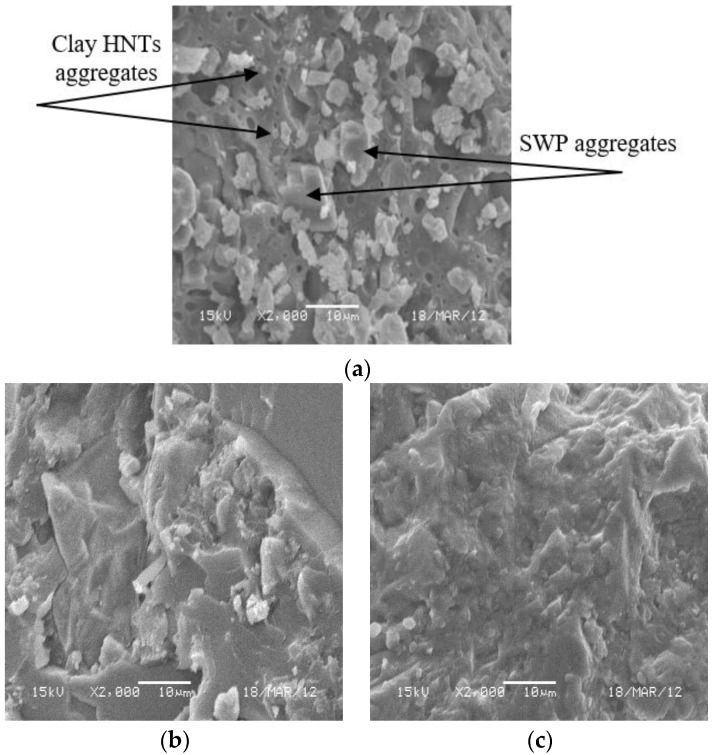
SEM micrographs of Granite/PMMA/clay HNTs nanocomposites impregnated with different SSF concentrations (**a**) 10 wt %, (**b**) 60 wt % compared to (**c**) natural granite.

SEM was used to study the microstructure of acrylic solid surfaces to know the effect of SSF concentration on their physical adhesion to PMMA nanocomposites; representative micrographs are shown in Figure 3. It is observed that, for the lowest filling concentration (10 wt % SSF), some voids are seen in addition to the flake-like structure resulting from the disconnections between SSF and PMMA nanocomposite matrix as illustrated by Figure 3a. Moreover, it is also found that reducing the SSF concentration increases the polymer free volume which promotes the aggregation of clay HNTs observed by the protrusions formed on the nanocomposite surface. The density of these protrusions and voids are noticeably lessened as the concentration of SSF is increased to 60 wt % (Figure 3b). Also, the adequate observation of this micrograph revealed that the interface between the inorganic and organic phases are indistinguishable. This means that the optimized PMMA/SSF nanocomposites may have improved mechanical performance due to the homogeneous distribution and complete enclosure of SSF in PMMA nanocomposite matrices. This can be ensured by comparing this result with the SEM micrograph obtained for natural granite presented in Figure 3c. 

**Figure 4:**
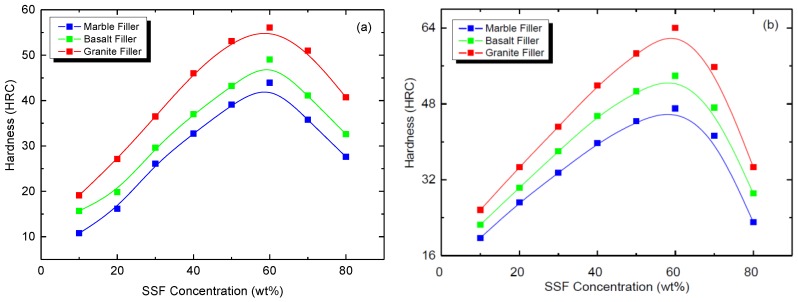
Effect of SSF concentration on the Rockwell hardness of acrylic solid surface nanocomposites (**a**) PMMA/SiO_2_/SSF and (**b**) PMMA/clay HNTs/SSF.

The effect of SSF concentration on the Rockwell hardness of PMMA/SiO_2_ and PMMA/clay HNTs nanocomposites is shown in Figure 4. It is noted that the hardness is increased by increasing the concentration of SSF up to 60 wt %, after which the hardness decreases. The reduction of the hardness beyond 60 wt % can be ascribed to the reduction of the polymer network connections at higher filling levels [20,21]. The maximum values of Rockwell hardness (HRC) obtained for PMMA nanocomposites entrapped with the investigated stones sludge fillers (SSF) are listed in Table 1. It is clearly observed that HRC values are larger for PMMA/clay HNTs due to the mechanical toughness of clay HNTs compared to hydrophilic nanosilica, reaching a maximum value when granite is used as fillers. This can be ascribed to the fact that the granite stone is harder than the basalt [22]. From this study, it can be revealed that the optimum concentration of SSF is 60 wt% to prepare PMMA/SSF nanocomposites for hard acrylic solid surface applications.

**Figure 5:**
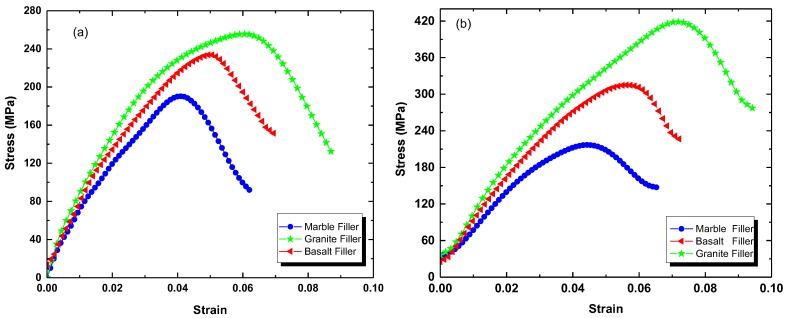
Flexural stress-strain curves of acrylic solid surface nanocomposites. (**a**) PMMA/SiO_2_/SSF and (**b**) PMMA/clay HNTs/SSF.

Figure 5 illustrates the flexural stress-strain curves of PMMA/SSF nanocomposites recorded according to ASTM (D 7264) at a strain rate of 1 mm/min. The modulus of elasticity *E* has been estimated according to Hook’s law relation [23].

*σ = E ε* (1)

where *σ* is the flexural stress, *ε* is the strain and *E* is the modulus of elasticity which is a measure of material resistance to deformation. The values of *E*, yield (maximum) stress *σ_m_*, and maximum strain *ε_m_* are listed in Table 2. From this study, it is clearly observed that the fine-powdered sludge stones enhanced the mechanical performance of the resulting nanocomposites. This can be ascribed to the increase of the chain stiffness of PMMA due to the adsorption of polymer chains onto the surface of the stone sludge fillers. Comparing these results to our previous work [5,7], it can be stated that the molecular motion and flexibility of the polymer chains is decreased by reducing the size of the filling stones due to the increase of polymer-mineral interaction that induces rigidity. 

**Figure 6:**
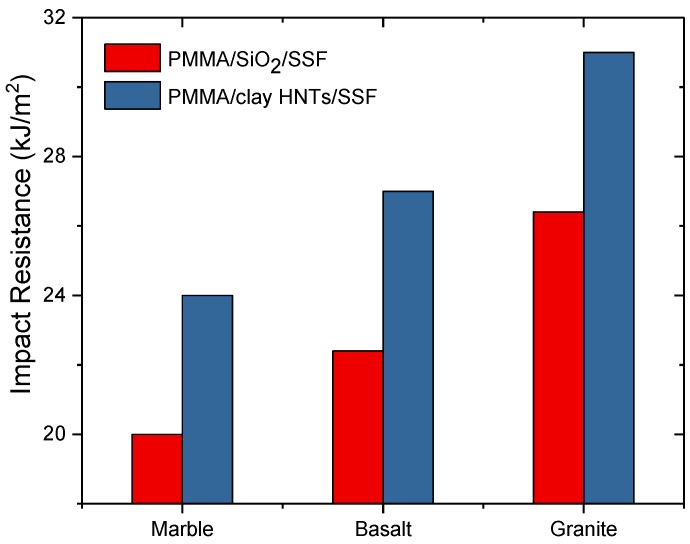
Effect of nanoparticle type on the impact strength of acrylic solid surface nanocomposite sheets.

The effect of nanoparticle type on the impact strength of acrylic solid surface nanocomposites is plotted in Figure 6. It is noticed that the impact strength for PMMA/SSF/clay HNTs is higher than that PMMA/SiO_2_/SSF nanocomposites. In addition, the maximum value of the impact resistance was 31 kJ/m^2^ obtained for granite stone fillers. This could be due to the high impact strength of granite compared to marble and basalt besides the excellent compatibility between PMMA/HNTs nanocomposite and granite sludge.

**Figure 7:**
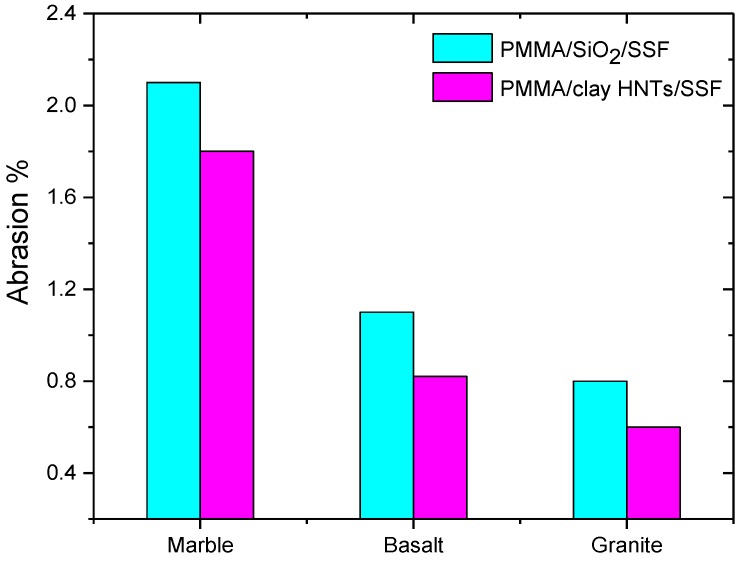
Effect of nanoparticle type on the abrasion percent of acrylic solid surface nanocomposite sheets.

The abrasion resistance of acrylic solid surface nanocomposites was estimated according to ASTM (D1044-08e1). Figure 7 illustrates the percentage weight loss due to surface abrasion for all the prepared acrylic solid surface nanocomposites. It is noticed that the abrasion resistance is increased for the nanocomposites containing clay HNTs compared to those containing nanosilica. This advantage is a result of clay HNTs which are excellent non-corrosive materials which provide a substantial increase in the scratch resistance [24]. It is also observed that granite sludge fillers are the best candidate for scratch resistant acrylic solid surface nanocomposites because granite stones are rich in highly scratch resistant crystalline phases [25].

**Figure 8:**
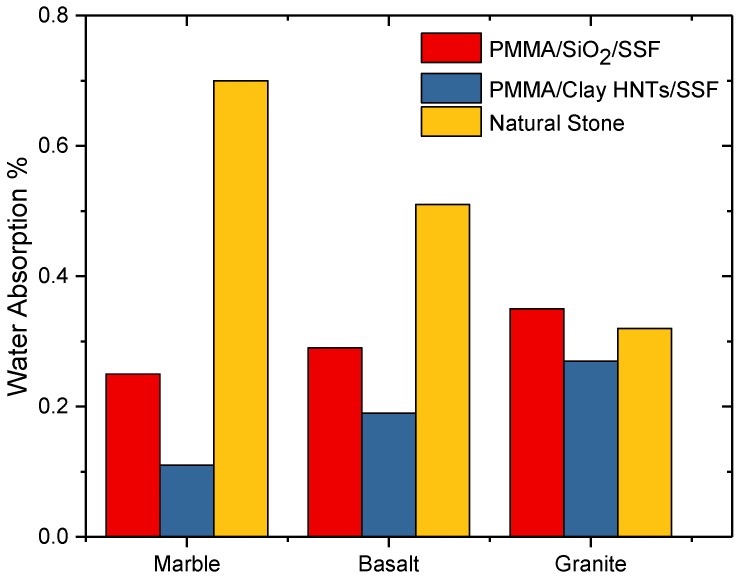
Effect of nanofiller types on the water absorption of acrylic solid surface nanocomposite sheets.

Water absorption was measured for solid surface nanocomposites after 24 h of soaking at normal atmospheric conditions according to ASTM (D570-98e1), the values are presented in Figure 8 compared to that of natural stones. It is clearly observed that the water absorption for acrylic solid surface nanocomposites based on clay HNTs is lower than those based on nanosilica. This can be attributed to the fact that polymer HNTs have a larger longitudinal size compared to spherical nanosilica and consequently a longer penetration distance in the resulting nanocomposite [5]. As a result of this phenomenon, the filler stones are protected from water diffusion after being completely saturated by PMMA nanocomposite solutions during the polymerization process. Moreover, the addition of both ATH and the cross-linking agents decreased the free volume of PMMA and prevented the presence of voids in the prepared matrix.

The density *ρ*, thermal conductivity *K*, and *DC* electrical conductivity *σ_dc_* were measured for acrylic solid surface sheets and compared to natural stones. Table 3 illustrates a remarkable decrease in the values of *K* and *σ_dc_* for acrylic solid surface nanocomposites due to the interfacial effects of the PMMA nanocomposite layers surrounding the stone filler particulates. This is favorable for the use of the prepared PMMA/SSF nanocomposites as a potential replacement of natural stones in interior building applications since there is good heat dissipation and high electrical resistivity caused by the interfacial resistance at the polymer–filler interfaces [26]. An additional important feature of the prepared acrylic solid surface systems is their low-density compared to natural stones. This recommends the ability of PMMA/SSF nanocomposites to be manufactured in a wide range of low densities and strengths by changing the concentration of the filling stone. Moreover, the light weight means the products are easy processed and transported, and subsequently cost-effective [5,6,7].

**Figure 9:**
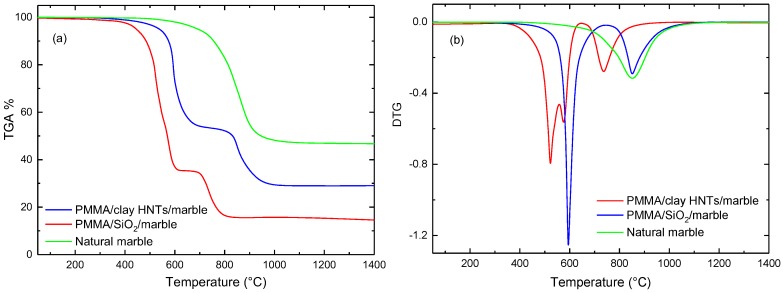
Effect of nanofiller type on thermogravimetric analyzer (TGA) (**a**) and derivative of the thermograms (DTG) (**b**) curves of marble solid surface nanocomposites (compared to natural marble).

**Figure 10:**
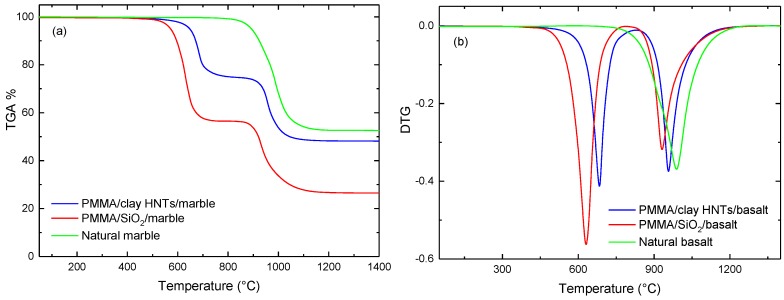
Effect of nanofiller type on TGA (**a**) and DTG (**b**) curves of Basalt solid surface nanocomposites (compared to natural basalt).

**Figure 11:**
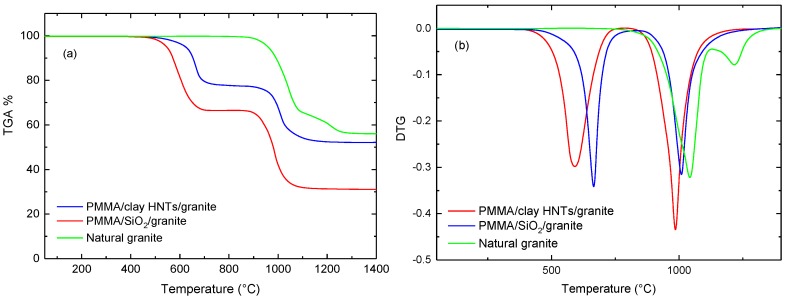
Effect of nanofiller type on TGA (**a**) and DTG (**b**) curves of Granite solid surface nanocomposites (compared to natural granite).

Thermogravimetric analysis was made at a heating rate 10 °C/min under a nitrogen atmosphere in the temperature range (25–1400 °C), the effect of nanoparticle type on the thermal stability of acrylic solid surfaces was studied by TGA curves shown in Figure 9, Figure 10 and Figure 11. It is observed that all TGA curves of acrylic solid surfaces have two phase transformation stages while natural stones exhibit one-step. The weight residues (W %) of the acrylic solid surfaces and natural stones after one-cycle heating were determined from the thermograms and listed in Table 4. It is noted that the values of W % obtained for clay filled acrylic solid surface nanocomposites are higher than those of nanosilica-based nanocomposites. In addition, the higher weight loss was obtained for marble filled acrylic solid surface nanocomposites. This can be ascribed to the fact that calcium carbonate (CaCO_3_), which is the main ingredient of marble, being thermally decomposed at (855 °C) as determined by the derivative of the thermograms (DTG) depicted in Figure 9, Figure 10 and Figure 11. The calculated DTG curves give valuable information to the mean temperatures of phase transformations for the two stages depicted previously by TGA thermograms [27]. For acrylic solid surface nanocomposites, two phase transformation temperatures were observed. The first, T^1st^ , corresponds to the mean temperature of phase decomposition of PMMA/nanocomposite matrix while the second, T^2nd^, corresponds to the thermal decomposition of the basic components of the filling stone [25]. Table 5 presents the effect of nanofiller type on the first and second phase transformation temperatures T^1st^ and T^2nd^ for acrylic solid surfaces sheets. It is observed that T^1st^ is increased for clay based granite samples, this can be explained by the excellent thermal stability of granites stones and clay HNTs [28]. This study reflects the thermal stability of acrylic solid surface nanocomposites and recommends them as promising candidates for acrylic solid surface applications, especially in hot climatic regions.

## 4. Conclusions

From this study, we concluded that PMMA/SiO_2_ and PMMA/clay HNTs nanocomposites can be considered as active host matrices for natural stone sludge for acrylic solid surface applications. Stone sludge fillers (SSF) enhanced the mechanical performance of PMMA nanocomposites which is likely to be used efficiently as an economical alternative to natural stones. The flexural strength and impact toughness measurements revealed the best mechanical performance for granite solid surface nanocomposites. Also, the excellent heat insulation and thermal stability for the acrylic solid surfaces sheets recommend their use in outdoor applications, especially in hot climates of KSA. More attention should be paid to sludge stone particulates which are of great interest due to their low cost, availability, physical properties, and suitability for wide range technological applications.

## Figures and Tables

**Table 1:** The maximum values of Rockwell hardness (HRC) obtained for PMMA nanocomposites entrapped with stone sludge fillers (SSF).

Stone	PMMA/Nano SiO_2_	PMMA/Clay HNTs
Marble	44.07	47.04
Basalt	49.40	53.92
Granite	56.06	64.02

**Table 2:** Effect of nanoparticle type on the flexural strength properties of PMMA/SSF nanocomposites.

Sample	*E* (10^3^ MPa)		*σ_m_* (MPa)		*ε_m_* (%)	
	Nano SiO_2_	Clay HNTs	Nano SiO_2_	Clay HNTs	Nano SiO_2_	Clay HNTs
Marble	4.72	5.50	190.10	218.00	4.16	4.54
Basalt	4.91	5.80	234.00	317.00	5.03	5.80
Granite	5.44	5.92	255.80	417.00	6.19	7.30

**Table 3:** The density, *ρ*, thermal conductivity, *K*, and electrical conductivity, *σ_dc_*, for optimized artificial stone sheets based on PMMA/SSF/clayHNTs nanocomposites compared to natural stones.

Stone	*ρ* (g/cm^3^)		*K* (cal/cm s °C)		*σ_dc_* (Ω^−1^ cm^−1^)	
	Natural	Artificial	Natural	Artificial	Natural	Artificial
Marble	2.56	1.57	6.51 × 10^−3^	2.82 × 10^−4^	1.21 × 10^−14^	1.20 × 10^−19^
Basalt	3.02	1.95	7.80 × 10^−3^	3.03 × 10^−4^	3.71 × 10^−7^	3.41 × 10^−18^
Granite	2.78	1.63	9.53 × 10^−3^	4.61 × 10^−4^	2.06 × 10^−9^	2.4 × 10^−17^

**Table 4:** The weight residues, W %, for the prepared artificial stones and natural stones after heating to 1400 °C in a nitrogen atmosphere.

Sample	PMMA/Nano SiO_2_	PMMA/Clay HNTs	Natural
Marble	15	29	47
Basalt	26	48	52
Granite	31	52	56

**Table 5:** The first and second phase transformation temperatures T^1st^ and T^2nd^ for the prepared artificial stones compared to natural stones.

Sample	PMMA/Nano SiO_2_		PMMA/Clay HNTs		Natural	
	T^1st^ (°C)	T^2nd^ (°C)	T^1st^ (°C)	T^2nd^ (°C)	T^1st^ (°C)	T^2nd^ (°C)
Marble	522	736	594	852	-	855
Basalt	630	932	685	958	-	980
Granite	592	985	665	1010	1042	1219
